# Association of subclinical atherosclerosis with echocardiographic indices of cardiac remodeling: The Framingham Study

**DOI:** 10.1371/journal.pone.0233321

**Published:** 2020-05-15

**Authors:** Cecilia Castro-Diehl, Rebecca J. Song, Gary F. Mitchell, David McManus, Susan Cheng, Ramachandran S. Vasan, Vanessa Xanthakis

**Affiliations:** 1 Department of Medicine, Section of Preventive Medicine and Epidemiology, Boston University School of Medicine, Boston, Massachusetts, United States of America; 2 Department of Epidemiology, Boston University School of Public Health, Boston, MA, United States of America; 3 Cardiovascular Engineering, Inc, Norwood, MA, United States of America; 4 Department of Medicine, University of Massachusetts Medical School, Worcester, MA, United States of America; 5 Smidt Heart Institute, Cedars-Sinai Medical Center, Los Angeles, CA, United States of America; 6 Boston University’s and National Heart, Lung and Blood Institute’s Framingham Heart Study, Framingham, MA, United States of America; 7 Department of Medicine, Section of Cardiology, Boston University School of Medicine, Boston, Massachusetts, United States of America; 8 Department of Biostatistics, Boston University School of Public Health, Boston, MA, United States of America; Nagoya University, JAPAN

## Abstract

**Background:**

It is well established that coronary artery disease progresses along with myocardial disease. However, data on the association between coronary artery calcium (CAC) and echocardiographic variables are lacking.

**Methods and results:**

Among 2,650 Framingham Study participants (mean age 51 yrs, 48% women; 40% with CAC>0), we related CT-based CAC score to left ventricular (LV) mass index (LVMi), LV ejection fraction (LVEF), E/e’, global longitudinal strain (GLS), left atrial emptying fraction (LAEF), and aortic root diameter (AoR), using multivariable-adjusted generalized linear models. CAC score (independent variable) was used as log-transformed continuous [ln(CAC+1)] and as a categorical (0, 1–100, and ≥101) variable. Adjusting for standard risk factors, higher CAC score was associated with higher LVMi and AoR (β_LVMI_ per 1-SD increase 0.012, β_AoR_ 0.008; *P*<0.05, for both). Participants with 1≤CAC≤100 and those with CAC≥101 had higher AoR (β_AoR_ 0.013 and 0.020, respectively, *P* = 0.01) than those with CAC = 0. CAC score was not significantly associated with LVEF, E/e’, GLS or LAEF. Age modified the association of CAC score with AoR; higher CAC scores were associated with larger AoR more strongly in older (>58 years; β_AoR_0.0042;*P*<0.007) than in younger (≤58 years) participants (β_AoR_0.0027;*P*<0.03).

**Conclusions:**

We observed that subclinical atherosclerosis was associated with ventricular and aortic remodeling. The prognostic significance of these associations warrants evaluation in additional mechanistic studies.

## Introduction

Coronary artery calcium (CAC) determined by computed tomography is a marker of subclinical atherosclerosis,[1] a non-invasive measure of coronary artery disease,[2] and a strong predictor of future coronary heart disease (CHD).[3] A CAC score of zero has been associated with a very low risk of obstructive CHD,[4] and middle-aged adults with a CAC score as low as 1–19 are shown to have higher risk of CHD events compared to those with CAC = 0 over 10 years of follow-up.[2] Morever, a high CAC score (CAC ≥ 400) is associated with higher risk of incident CHD and may trigger further investigations for its diagnosis and treatment,[4] while CAC scores in the intermediate range (>100 but <300) may also justify noninvasive follow-up examinations for CHD risk prediction and management.[4]

The presence and severity of CAC have been linked to poor myocardial perfusion among individuals without overt clinical CHD.[5] In a recent meta-analysis of six studies involving 2,123 symptomatic and asymptomatic patients with suspected coronary artery disease, the prevalence of inducible myocardial ischemia rose with a gradual increase of the CAC score.[6]

Edvardsen et al reported that regional CAC was associated with regional myocardial dysfunction, as measured by cardiac magnetic resonance imaging, among individuals without prevalent heart disease.[7] Subclinical atherosclerosis may also lead to left ventricle (LV) remodeling manifested by increased LV mass (LVM),[8] and worse LV diastolic function.[9] The precise mechanisms involved in the LV remodeling and diastolic disfunction are not clear. We could elucidate that the increase in wall thickness, a feature of greater LV mass, may result in impaired myocardial relaxation, increased ventricular stiffness, and increased LV filling pressure[10,11] and that changes in aortic stiffness may also lead to LV remodeling. However, we cannot discard the possibility that a hypertrophied ventricle may produce damaging pressure swings that promote CAC or that aortic stiffness may simultaneously lead to increased CAC and LV remodeling. Changes in LV structure and function may also potentially affect left atrial (LA) structure and function.[12] LA remodeling can be identified by increased LA dimensions and reduced total LA emptying fraction (LAEF). Subtle changes in the myocardial strain can also be observed in subclinical atherosclerosis, as detected by decreased (in negative value) global circumferential and longitudinal strain (GCS, GLS).[13] In addition of the above physiological and structural changes, the portion of the beginning of the aorta at the aortic annulus, aortic root, will go through remodeling over the lifecourse and these changes can also precede cardiovascular diseases.[14]

There are limited reports of the relation between CAC and echocardiographic variables. While some studies have observed an association of higher CAC with higher LVMI,^8^ higher E/e’,[15] and larger left atrium,[16] other studies did not observe similar relations.[17]

The overall evidence to date suggests that coronary disease interacts and progresses along with the incipient development of myocardial disease, even in the absence of symptoms. Accordingly, we hypothesized that participants free of overt CHD but with higher CAC score values have worse indices of cardiac and aortic remodeling (higher LVMi, GLS and E/e’ ratio and lower LVEF, LAEF, and aortic root diameter [AoR]) than participants with lower values of CAC score.

## Methods

### Study sample

Details of the design of the Offspring and the Third Generation cohorts of the Framingham Heart Study have been reported previously.[18] Participants in the Offspring cohort underwent routine transthoracic echocardiography during the eighth examination cycle (2005–2008) and computerized tomography scanning of their coronary arteries at the end of the eight examination cycle (2008–2011). Participants in the Third-Generation cohort underwent tomography scanning and transthoracic echocardiography during their first examination cycle (2002–2005). For the Offspring cohort, covariates and echocardiographic variables were measured approximately 3 years before the assessment of CAC (covariates and echocardiographic variables were assessed between 2005–2008 whereas CAC was assessed between 2008–2011). For the Third generation cohort, all variables (CAC, covariates, and echocardiographic variables) were assessed contemporaneously.

For the present investigation, there were 3528 eligible participants (**S1 Fig**). We excluded participants if they had prevalent myocardial infarction and/or congestive heart failure (n = 57), were not in sinus rhythm (n = 129), or had incomplete data on echocardiographic indices (n = 692), resulting in a final sample size of 2650 participants with available CAC measurements and data on echo indices of interest for the present investigation. Following standardized protocols, participants provided a detailed medical history and underwent physical examination and laboratory tests for cardiovascular disease risk assessment. The study was approved by the Institutional Review Board of Boston University Medical Center, and all participants gave written informed consent.

### Coronary Artery Calcium (CAC) assessment

CAC was assessed by chest CT using an eight-slice multidetector CT scanner (LightSpeed Ultra; General Electric Milwaukee, WI). All participants were scanned twice according to a sequential scan protocol described previously.[19] Experienced readers examined independently the scans offline to identify and quantify coronary calcification, as reported previously.[19,20] The amount of CAC was calculated using the Agatston score, [20,21] and the scores from two scans were averaged. We reported excellent reproducibility in the FHS cohorts.[20]

### Echocardiographic measurements

Transthoracic echocardiography with color flow and tissue Doppler imaging was performed by experienced sonographers on all attendees using a Hewlett-Packard Sonos 5500 Ultrasound machine (Phillips Healthcare, Andover, MA) and analyzed using an offline system (DigiView System Software (ver. 3.7.9.3, Digisonics Inc., Houston, Texas, USA)). Linear cardiac dimensions, and annular displacement and velocities were measured. Echocardiographic images were digitized and stored in Digital Imaging and Communications in Medicine (DICOM) format. Speckle tracking echocardiographic strain analysis of the LV based on 2D images was also performed using an offline image analysis program (2D Cardiac Performance Analysis v1.1, TomTec Imaging Systems, Unterschleissheim, Germany). We categorized the echocardiographic indices as primary (standard echocardiographic indices for which there are published associations with several outcomes) and secondary (additional novel echocardiographic indices).

Two independent readers evaluated all echocardiographic measurements to determine agreement. The reproducibility of the echocardiographic measurements was excellent.[22] A validated test was performed for assessing intra- and inter-reader variability. Correlation within readers (for first two readings) was 0.917 to 0.983, and between readers (for first reading) was 0.901 to 0.967. FHS has reported no major systematic bias across exams due to change of equipment.[23]

### Primary echocardiographic indices

For the present investigation, we used the following primary echocardiographic indices: left ventricular (LV) mass indexed by body surface area (LVMi), LV ejection fraction (LVEF), aortic root diameter (AoR), left atrial emptying fraction (LAEF), E/e’, and global longitudinal strain (GLS).

LV mass (LVM) was derived from measurements of end-diastolic LV septal wall thickness (SWT), posterior wall thickness (PWT), LV end-diastolic diameter (LVDD) and LV end-systolic diameter (LVSD)[24] using the American Society of Echocardiography (ASE)-recommended formula,[25] and then indexed to body surface area. LVEF was calculated from the LV volumes, LV end-diastolic (LVEDV) and LV end-systolic volume (LVESV), derived from linear measurements as previously described.[26] AoR was measured using the leading-edge technique as previously described.[27] LAEF, an indicator of LA total emptying, was calculated based on minimum (min) and maximum (max) LA volumes obtained at mitral valve closure (systole) and just before mitral valve opening (after LA contraction), respectively, using the following formula ([LAmax—LAmin]/LAmax)*100.[28,29] LAmax and LAmin were obtained using the recommended Simpson’s biplane summation of disks.[30] LAmax and LAmin were calculated by averaging LAmax and LAmin measurements from the apical two- and four chamber views.[31] We included LAEF only for the Offspring cohort (n = 885 participants), in this analysis, because LAEF data were not available for participants in the Third Generation cohort. Early transmitral Doppler flow velocity (E wave) and tissue Doppler assessment of peak early diastolic tissue velocity of the lateral mitral annulus (e’) were measured by pulse-wave Doppler from the apical four-chamber view.[32] The ratio of E/e’ was used as a surrogate for LV filling pressure. Global longitudinal strain (GLS) represents the myocardial shortening in the long axis plane and was obtained using apical views. In the present investigation, GLS was calculated as the average peak longitudinal strain measured in 12 regions in the apical two- and four chamber views.[33] Since GLS values are negative, a value less than −20% is likely considered to be normal[13] whereas GLS values closer to zero indicate worse cardiac function.

### Secondary echocardiographic indices

The following secondary echocardiographic indices were also analyzed: LVDD, LV wall thickness (LVWT), LV wall motion (LVWM) abnormality, mitral annular plane systolic excursion (MAPSE), global circumferential strain (GCS), and LV longitudinal segment synchrony (LSS).

LVWM is an indicator of global LV function. MAPSE is an indicator of long axis LV function and was obtained by measuring the mitral annular displacement.[34] GCS is a measure of global deformation and represents the myocardial shortening in the short axis.[13] LSS was calculated as the standard deviation (SD) of time-to-peak systolic longitudinal strains[35] generated from speckle-tracking echocardiography.

### Covariates

For the present investigation, we used the following covariates: age, sex, cigarette smoking (yes/no) assessed by questionnaire, body mass index, systolic and diastolic blood pressure (measured by a study physician using a standardized protocol), self-reported use of blood pressure-lowering medications, diabetes status (defined as fasting glucose ≥126 mg/dL or treatment with either insulin or a hypoglycemic agent), the ratio of serum total cholesterol over high density-lipoprotein, use of lipid-lowering medication, and physical activity.

Each FHS clinic examination included anthropometry, blood pressure measurements, and electrocardiography. All procedures followed a standardized protocol. Height and weight were measured during the examination and body mass index (BMI) was calculated as weight in kilograms divided by height in meters squared (kg/m^2^). Heart rate was derived from electrocardiography. Participants also underwent phlebotomy after overnight fast for measurement of glucose, lipids and creatinine. After blood specimens were collected, the plasma was separated. HDL was separated by precipitating the other lipoproteins with heparin-manganese chloride using a modification of the technique described by Burstein et al.[36] Cholesterol concentrations were determined by the Abell-Kendall method. Triglycerides were determined by a modification of the Keesler-Lederer method.[37] Glucose was measured in whole blood and was determined using the method of Somogyi-Nelson.[38]

We estimated the physical activity index using self-reported hours of sleep, sedentary, light, moderate and vigorous activity from the FHS clinic examination. The physical activity index was calculated using the following equation: [sleep time + (1.1*sedentary time) + (1.5*slight activity) + (2.4*moderate activity) + (5*heavy activity)].

### Statistical analysis

We natural logarithmically-transformed values of LVMi, LVEF, AoR and E/e’ to normalize their skewed distributions. Because CAC scores also showed a skewed distribution, and many participants had a CAC score of zero, we added 1 to all CAC values and then logarithmically-transformed the new values [ln(CAC+1)].

We used generalized linear models to relate CAC score (independent variable) to echocardiographic indices (dependent variables; separate model for each), adjusting for age, sex, BMI, smoking (yes/no), systolic and diastolic blood pressure, current use of hypertensive medications (yes/no), diabetes (yes/no), total cholesterol/HDL ratio, use of lipid-lowering medication, and physical activity, and accounted for relatedness among individuals. We also accounted for multiple comparisons by using false discovery rates, with a q value<0.05 indicating statistical signficance.[39] We also created restricted cubic splines to assess the linearity of the association between the echocardiographic variables and CAC.

We first examined CAC score as a continuous variable, and then as a categorical variable (0, 1–100 and ≥101; cutpoints published in the literature) with CAC = 0 serving as the reference category.[40] We also tested for effect modification by age and sex by including corresponding interaction terms in the models. A P-value of <0.05 for the interaction was considered statistically significant.

## Results

Characteristics of the study sample are shown in **Table 1**. Our sample included young, middle-aged and older adults (48% women) with a BMI in the overweight range. The prevalence of CAC >0 was 40% in our sample. More men than women had CAC>0 (49% vs 29%) and CAC ≥101 (20% vs 9%). Participants’ characteristics by absence vs. presence of CAC are shown in **S1 Table.** Participant characteristics by cohort (Offspring vs. Third Generation) are shown in **S2 Table.**

**Table 1:** Characteristics of study sample.

	Men (n = 1369)	Women (n = 1281)
**Clinical Characteristics**		
Age, y	50±12	53±11
Height, cm	177±7	163±6
Weight, kg	87±14	71±16
Body mass index, kg/m^2^	27.9±4.1	26.7±5.7
Smoking, %	10	12
Systolic blood pressure, mm Hg	123±14	120±16
Diastolic blood pressure, mm Hg	78±9	73±9
Hypertension, %	31	29
Hypertension treatment, %	20	22
Heart rate, bpm	58±9	61±9
Diabetes, %	6	4
Serum creatinine, mg/100ml	0.9±0.2	0.7±0.2
Total cholesterol, mg/dL	191±34	194±35
HDL cholesterol, mg/100ml	48±13	63±18
LDL cholesterol, mg/100ml	118±31	110±31
Triglycerides, mg/100ml	106(73–156)	88(65–126)
Lipid-lowering medication use, %	21	16
Phyical activity index	38±8	36±6
**Coronary artery calcification (CAC)**		
Coronary Artery Calcium Score	0 (0–56)	0 (0–3)
Prevalence of CAC Score, %		
0	51	71
1–100	29	20
≥101	20	9
**Echocardiographic variables**		
*Primary*		
LV Mass Index, g/m^2^	91 (81,102)	75 (67,85)
LV Ejection Fraction, %	65 (62,68)	67 (64,71)
Aortic Root, cm	3.4 (3.2,3.6)	3.0 (2.8,3.2)
LA Emptying Fraction, %	48.3 (46.7,49.6)	48.3 (46.3,9.9)
E/e’ratio	5.7 (4.9,6.7)	6.4 (5.4,7.7)
GLS, %	-19.0 (-20.8,-17.3)	-21.2 (-23.1,-19.3)
*Secondary*		
LV Diastolic Diameter, cm	5.2 (4.9,5.4)	4.7 (4.5,5.0)
LV Wall thickness, cm	2.0 (1.9,2.1)	1.7 (1.6,1.8)
Wall motion abnormality, %	2.0	0.9
MAPSE, cm	1.6 (1.4,1.7)	1.6 (1.4,1.7)
GCS, %	-28.9 (-31.8,-25.8)	-30.7 (-34.2,-27.5)
LSS, msec	99 (75,117)	96 (59,116)

All values shown are mean ± standard deviation or median (Q1,Q3), unless otherwise specified

LAEF is computed among Offspring participants only

LV = Left Ventricular, LA = Left Atrial GLS = Global longitudinal strain, MAPSE = Mitral Annular Plane Systolic Excursion, GCS, Global circumferential strain, LSS, Longitudinal segmental synchrony

Adjusting for covariates, higher CAC values were associated with higher levels of LVMi and AoR. We did not observe an association between continuous CAC and LVEF, LAEF, E/e’ or GLS (**Table 2**). Participants with CAC score of 1–100 and those with CAC score ≥101 had higher AoR values compared to the referent group with CAC score of zero. We did not observe an association between coronary artery calcium and any of the secondary echocardiography variables selected (data not shown).

**Fig 1:**
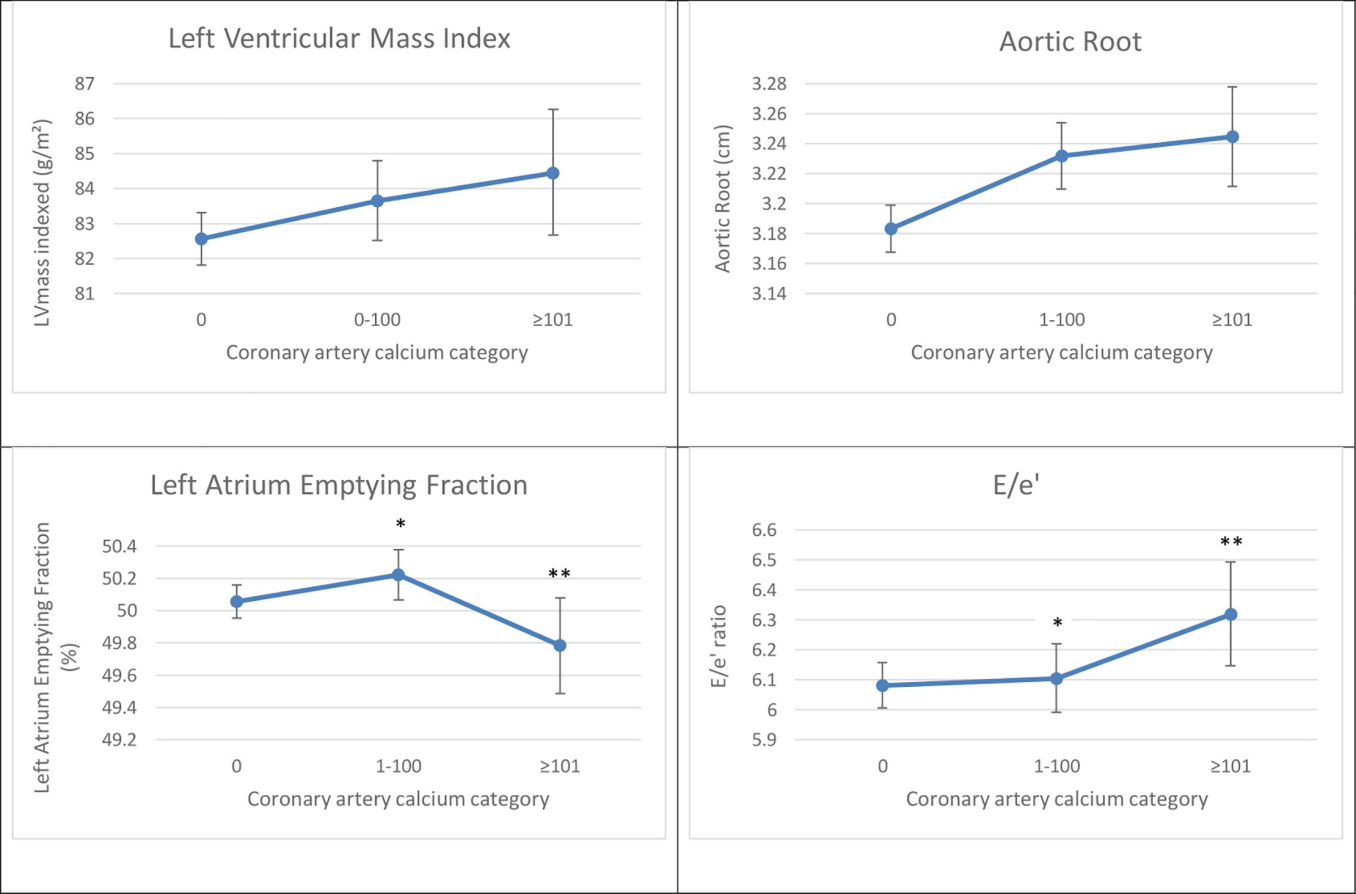
Least square means of echocardiographic indices by CAC category. Least-Square Means were adjusted for age, sex, smoking, systolic blood pressure, current use of hypertensive medications, diabetes, total cholesterol/HDL ratio, lipid-lowering medication and physical activity index. *Comparison versus CAC = 0, p<0.05 **Comparison versus CAC = 1–100, p<0.05.

**Fig 1** shows multivariable-adjusted least square mean (LSM) values for LVMi, AoR, LAEF, and E/e’ according to CAC score category.

**Table 2:** Associations between CAC and echocardiographic indices.

	LVMi (log(g/m^2^))		LVEF (log(%))		AoR (log(cm))		LAEF^§^ (%)		E/e′ (log(E/e′ ratio))		GLS (%)	
CAC score	Estimate (SE)	*P*-value	Estimate (SE)	*P*-value	Estimate (SE)	*P*-value	Estimate (SE)	*P*-value	Estimate (SE)	*P*-value	Estimate (SE)	*P*-value
**CAC as continuous variable**												
CAC*	0.012 (0.004)	**0.048**	−0.002 (0.002)	0.54	0.008 (0.002)	**0.005**	−0.220 (0.092)	0.06	0.009 (0.006)	0.24	0.049 (0.069)	0.61
**CAC as categorical variable**												
CAC = 0 (n = 1602)	Referent		Referent		Referent		Referent		Referent		Referent	
CAC = 1–100^†^ (n = 659)	0.014 (0.008)	0.20	0.004 (0.004)	0.55	0.013 (0.004)	**0.01**	-0.090 (0.190)	0.69	0.001 (0.011)	0.94	0.098 (0.140)	0.61
CAC ≥ 101^†^ (n = 389)	0.020 (0.012)	0.23	−0.006 (0.006)	0.44	0.020 (0.006)	**0.01**	−0.654 (0.265)	0.06	0.031 (0.016)	0.14	0.079 (0.193)	0.71
*P-*trend^‡^		**0.048**		0.59		**<0.001**		**0.02**		0.11		0.57

* Estimates are per 1-SD increase in ln(CAC+1)

^†^ reference group, CAC = 0 (n = 1602)

^‡^ trends across the CAC categories.

^**§**^ LAEF estimate among Offspring participants only.

All models were adjusted for age, sex, smoking (yes/no), systolic blood pressure, diastolic blood pressure, current use of hypertensive medications (yes/no), diabetes (yes/no), total cholesterol/HDL ratio, lipid-lowering medication, physical activity index, and body mass index. *P*-values are FDR *q*-values.

**Fig 2:**
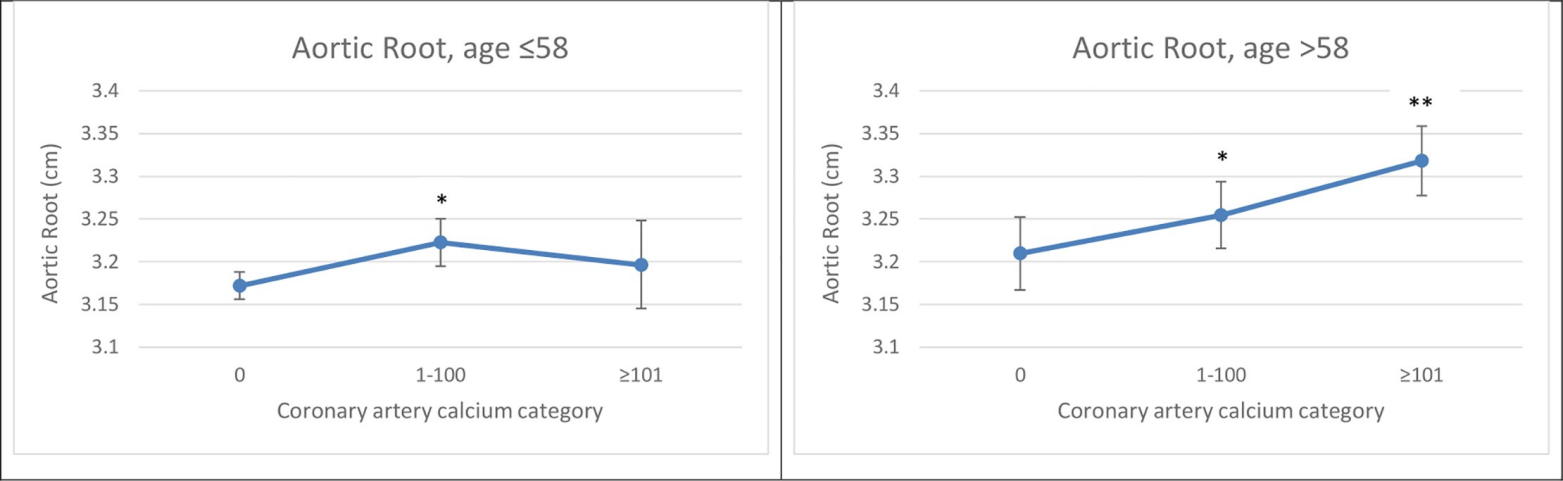
Associations of CAC with AoR stratified by median age. Least-Square Means were adjusted for age, sex, smoking, systolic blood pressure, diabetes, total cholesterol/HDL ratio, current use of hypertensive medication, lipid-lowering medication and physical activity index. *Comparison versus CAC = 0, p<0.05 **Comparison versus CAC = 1–100, p<0.05.

Additionally, we observed effect modification by age, but not by sex, for the association of CAC score with AoR (*P* for interaction = 0.049). In stratified analysis, the association of CAC values varied with age, with the association being stronger among older participants (age >58 yrs) than in younger participants. **Fig 2** shows multivariable-adjusted LSM values for AoR by CAC score category, stratified by median age.

When testing for non-linearity of associations using multivariable-adjusted restricted cubic splines relating the log-transformed CAC to the primary echocardiographic measures, LVMi and AoR showed linear associations with log-transformed CAC (**S2 Fig**).

## Discussion

### Principal findings

In our large community-based sample of middle-aged and older participants without congestive heart failure or myocardial infarction, we observed that higher CAC scores were associated with higher values of LVMi and AoR consistent with potential adverse effects of subclinical atherosclerosis on myocardial, chamber, and aortic remodeling. Of note, the effect sizes were modest and their clinical significance, if any, is unknown.

#### CAC score and LV structure and function

The positive association between CAC and LVMi is consistent with previous studies. For example, Gardin et al reported that a 5-year increase in LVM is associated with higher odds of CAC.^8^ However, both CAC and echocardiographic variables were measured 10 years apart, rendering the directionality of this association unclear. Other investigations reporting a direct association between CAC scores and LVMi have used smaller sample sizes and included elderly participants or participants with suspected coronary artery disease, who are at much higher risk of both CAC and LV hypertrophy.[15, 41] In a most recent investigation from the CARDIA study, with 5115 white and black participants who were healthy at baseline, higher CAC scores among middle-aged people were associated with higher LV mass adjusting for standard cardiovascular risk factors.[9] The exact mechanisms involved in the association between CAC score and LVMi are not known.

In our sample, we did not observe an association of CAC with E/e’. However, in a study by Osawa et al,[15] participants with CAC≥400 had significantly higher E/e’ than those with CAC = 0–9, 10–99, or 100–399. However, the participants were older (70±8 years) than in our sample (52±12 years), and the mean E/e’was higher (12±6) than that in our sample (median E/e’ = 5.7 [4.9–6.7] for men and 6.4 [5.4–7.7] for women). In our investigation, participants did not have CAC values close to 400, contrary to the aforementioned study[15]. Furthermore, other investigations have reported higher CAC scores being associated with higher LV filling pressures among healthy participants[9] as well as among those with suspected coronary artery disease.[42] However, in a retrospective study of 349 asymptomatic patients with a median CAC score of 14 (Q1, Q3: 0, 136), no association between CAC score and diastolic dysfunction was observed,[17] but, in this group of participants, higher CAC score was associated with higher LA volume index, a surrogate of diastolic dysfunction.

#### CAC score and aortic remodeling

We observed a positive association between CAC and aortic root diameter (AoR) values and the association was stronger among older (compared to younger) participants. Subclinical atherosclerosis as assessed by CAC may be associated with enlargement of the AoR. It is conceivable that CAC parallels aortic atherosclerosis, and the latter is associated with compensatory enlargement of the root as a result of atherosclerotic burden (Glagov phenomenon[43]). Although there are a few studies that have observed an association between CAC and aortic root calcification using CT,[44] to the best of our knowledge, studies investigating the relation between CAC and AoR measured by echocardiography are lacking. Medial artery calcification is associated with advanced age and this type of calcification is linked to arterial stiffness;[14] this may explain why we observed a stronger association between CAC and AoR among older participants (those with a median age >58).

We did not observe an association between CAC and LVEF, LAEF, E/e’ or GLS, indicators of systolic and diastolic chamber and myocardial function, perhaps because the prevalence of CAC in our sample was relatively low. We did not observe significant associations between CAC and some of the echocardiographic variables perhaps due to the fact that our sample had a very low prevalence of CAC >300.

#### Strengths and limitations

Our study sample had a wide age range, with a high percent being hypertensive. We conducted a comprehensive assessment of cardiovascular risk factors and several indicators of cardiac remodeling to detect associations between CAC burden and cardiac and aortic remodeling. Previous investigations have evaluated only a limited number of echocardiographic variables.

However, our study has several limitations. First, we cannot infer causality of any of the observed associations, given the cross-sectional nature of design. Second, because the study participants were predominantly white, largely middle-class, and middle-aged (median age 58 years), our findings cannot be generalized to other age groups or ethnicities; additional studies of multiethnic samples are warranted. Third, CAC values are highly correlated with atherosclerotic plaque area,[1] but not all atherosclerotic plaques contain calcium. Therefore, the predominantly low coronary calcium levels in our sample may have limited our statistical power to detect associations of atherosclerotic plaques and cardiac remodeling. Fourth, some of the observed associations may be statistically significant but given the small magnitude of effect sizes, we understand that they may not have a high clinical importance. Fifth, the Third generation participants did not have measurements on LAEF, so the sample size was smaller for that part of the analysis. Finally, the magnitude of the effect sizes are small and we cannot rule out measurement error.

## Conclusion

Our results provide suggestive evidence of an association between subclinical atherosclerosis and cardiac and aortic remodeling in individuals free of congestive heart failure and myocardial infarction. These findings indicate that interactive as well as parallel development of coronary vascular and myocardial disease may be a common phenomenon among aging individuals in our community-based sample. Additional mechanistic studies are warranted to further clarify the pathophysiological basis of the observed associations and the prognostic significance for the use of CAC on cardiac and aortic remodeling, if any, in more diverse populations.

## Supporting information

S1 Fig Determination of the eligibility criteria. (DOCX)

S2 Fig Restricted cubic splines. Multivariable-adjusted restricted cubic splines of log-transformed CAC by log-transformed LVMi (log(g/m^2^)), log-transformed AoR (log(cm)) with knots placed at 50^th^, 75^th^ and 95^th^ percentile values.
(DOCX)

S1 Table Characteristics of study sample by CAC status. (DOCX)

S2 Table Characteristics of study sample by cohort. (DOCX)
