# Supplementary material for: Association of subclinical atherosclerosis with echocardiographic indices of cardiac remodeling: The Framingham Study
Source: PLoS One. 2020 May 15;15(5):e0233321. doi: 10.1371/journal.pone.0233321 (PMC7228064; doi:10.1371/journal.pone.0233321)
Supplement: S1 Fig — (DOCX) [file pone.0233321.s001.docx]

**Figure S1.** Determination of the eligibility criteria

Participants with valid CAC score and echocardiographic data n = 2836

Participants with valid CAC score

n = 3528

No echocardiography data

excluded:

n= 692 participants

No sinus rhythm

excluded:

n = 129 participants

Prevalent MI and/or CHF excluded:

n = 57 participants

Final sample

n = 2650 participants
