# Supplementary material for: Association of subclinical atherosclerosis with echocardiographic indices of cardiac remodeling: The Framingham Study
Source: PLoS One. 2020 May 15;15(5):e0233321. doi: 10.1371/journal.pone.0233321 (PMC7228064; doi:10.1371/journal.pone.0233321)
Supplement: S2 Fig — Multivariable-adjusted restricted cubic splines of log-transformed CAC by log-transformed LVMi (log(g/m2)), log-transformed AoR (log(cm)) with knots placed at 50th, 75th and 95th percentile values. (DOCX) [file pone.0233321.s002.docx]

| 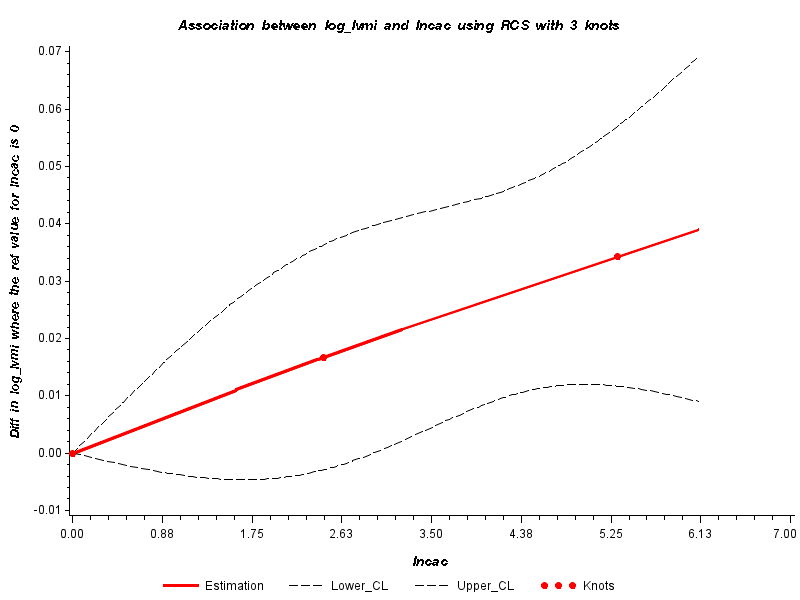  Log-transformed CAC by log-transformed LVMi (log(g/m^2^)) Test for non-linearity (*P*=0.89) |  |
| --- | --- |
| 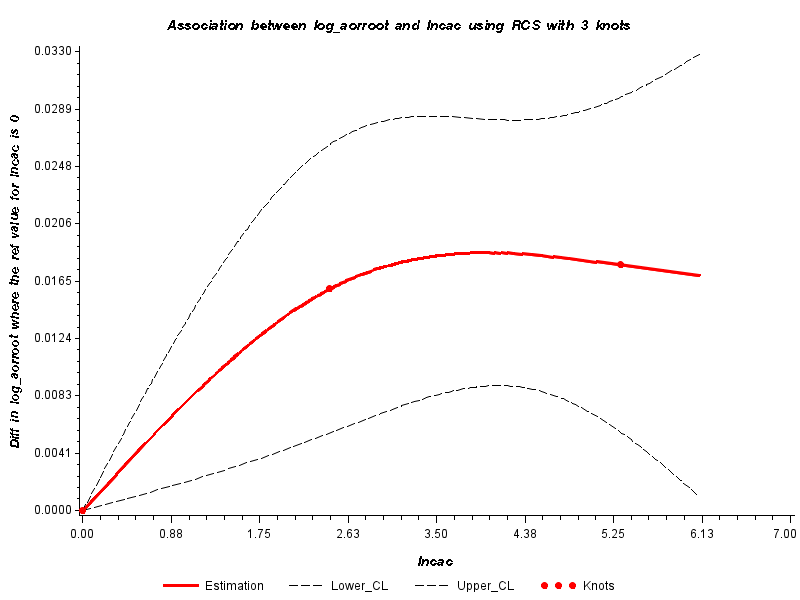  Log-transformed CAC by log-transformed AoR (log(cm))  Test for non-linearity (*P*=0.18) |  |

**Figure S2.** Restricted cubic splines

Multivariable-adjusted restricted cubic splines of log-transformed CAC by log-transformed LVMi (log(g/m^2^)), log-transformed AoR (log(cm)) with knots placed at 50^th^, 75^th^ and 95^th^ percentile values.

**Supplementary Figure S1**
