# Supplementary material for: Association of subclinical atherosclerosis with echocardiographic indices of cardiac remodeling: The Framingham Study
Source: PLoS One. 2020 May 15;15(5):e0233321. doi: 10.1371/journal.pone.0233321 (PMC7228064; doi:10.1371/journal.pone.0233321)
Supplement: S1 Table — (DOCX) [file pone.0233321.s003.docx]

**Table S1.** Characteristics of study sample by CAC status

|  | **CAC = 0 (n=1602)** | **CAC > 0 (n=1048)** |
| --- | --- | --- |
| **Cardiovascular Risk Factors** | | |
| Age, years | 47±9 | 58±12 |
| Women, % | 57 | 36 |
| Height, cm | 170±10 | 171±10 |
| Weight, kg | 78±17 | 82±17 |
| Body mass index, kg/m^2^ | 26.8±5.0 | 28.1±4.9 |
| Smoking, % | 11 | 11 |
| Systolic Blood Pressure, mm Hg | 118±14 | 127±16 |
| Diastolic Blood Pressure, mm Hg | 75±9 | 76±10 |
| Hypertension, % | 20 | 45 |
| Hypertension treatment, % | 12 | 34 |
| Heart Rate, bpm | 59±9 | 59±9 |
| Diabetes, % | 2 | 9 |
| Serum creatinine, mg/100ml | 0.8±0.2 | 0.9±0.3 |
| Total cholesterol, mg/dL | 192±34 | 193±35 |
| High Density Lipoprotein, mg/100ml | 57±17 | 53±16 |
| Low Density Lipoprotein, mg/100ml | 114±31 | 115±32 |
| Triglycerides, mg/100ml | 107±57 | 123±66 |
| Lipid-lowering medication use, % | 10 | 33 |
| Phyical activity index | 37±7 | 37±7 |
| **Echocardiographic Variables** | | |
| *Primary Variables (main outcomes)* |  |  |
| LV Mass Index, g/m^2^ | 80 (71,92) | 88 (77,101) |
| LV Ejection Fraction, % | 65 (62,68) | 66 (63,70) |
| Aortic Root, cm | 3.1 (2.9,3.4) | 3.4 (3.1,3.6) |
| LA Emptying Fraction, % | 49.2 (47.6,50.5) | 47.8 (46.1,49.3) |
| E/e' | 5.8 (5.0,6.9) | 6.3 (5.3,7.7) |
| GLS, % | -20.0 (-22.2,-18.2) | -19.7 (-21.8,-17.8) |
| *Secondary Variables* |  |  |
| LV Diastolic Diameter, cm | 4.9 (4.6,5.2) | 5.0 (4.7,5.3) |
| LV Wall thickness, cm | 1.8 (1.6,2.0) | 1.9 (1.8,2.1) |
| Wall motion abnormality, % | 0.6 | 2.8 |
| MAPSE, cm | 1.6 (1.4,1.7) | 1.5 (1.4,1.7) |
| GCS, % | -29.2 (-32.3,-26.2) | -30.5 (-34.2,-27.1) |
| LSS, msec | 101 (80,119) | 87 (54,112) |

Values shown are mean ± standard deviation or median (Q1, Q3) unless otherwise specified.

LAEF is computed among the Offspring cohort only

LV = Left Ventricular, LA = Left Atrial GLS = Global longitudinal strain, MAPSE = Mitral Annular Plane Systolic Excursion, GCS, Global circumferential strain, LSS, Longitudinal segmental synchrony
