# Supplementary material for: Association of subclinical atherosclerosis with echocardiographic indices of cardiac remodeling: The Framingham Study
Source: PLoS One. 2020 May 15;15(5):e0233321. doi: 10.1371/journal.pone.0233321 (PMC7228064; doi:10.1371/journal.pone.0233321)
Supplement: S2 Table — (DOCX) [file pone.0233321.s004.docx]

**Table S2.** Characteristics of study sample by cohort

|  | **Offspring (n=885)** | **Third Generation (n=1765)** |
| --- | --- | --- |
| **Cardiovascular Risk Factors** | | |
| Age, years | 65±9 | 45±6 |
| Women, % | 56 | 45 |
| Height, cm | 167±10 | 172±9 |
| Weight, kg | 78±16 | 80±17 |
| Body mass index, kg/m^2^ | 27.7±5.0 | 27.1±5.0 |
| Smoking, % | 7 | 13 |
| Systolic Blood Pressure, mm Hg | 118±14 | 127±16 |
| Diastolic Blood Pressure, mm Hg | 75±9 | 76±10 |
| Hypertension, % | 50 | 19 |
| Hypertension treatment, % | 42 | 11 |
| Heart Rate, bpm | 59±9 | 59±9 |
| Diabetes, % | 9 | 3 |
| Serum creatinine, mg/100ml | 0.9±0.3 | 0.8±0.2 |
| Total cholesterol, mg/dL | 190±36 | 194±34 |
| High Density Lipoprotein, mg/100ml | 58±17 | 54±17 |
| Low Density Lipoprotein, mg/100ml | 109±31 | 117±31 |
| Triglycerides, mg/100ml | 114±57 | 113±63 |
| **Echocardiographic Variables** | | |
| *Primary Variables (main outcomes)* |  |  |
| LV Mass Index, g/m^2^ | 85 (73, 99) | 82 (73, 94) |
| LV Ejection Fraction, % | 68 (63, 72) | 65 (62, 68) |
| Aortic Root, cm | 3.2 (3.0, 3.5) | 3.2 (3.0, 3.5) |
| LA Emptying Fraction, % | 48.3 (46.5, 49.8) | - |
| E/e' | 6.6 (5.5, 8.0) | 5.8 (5.0, 6.7) |
| GLS, % | -20.8 (-22.7, -18.9) | -19.6 (-21.7, -17.7) |

Values shown are mean ± standard deviation or median (Q1, Q3) unless otherwise specified.

LAEF is computed among the Offspring Cohort only

LV = Left Ventricular, LA = Left Atrial GLS = Global longitudinal strain
